# Bioinspired magnetic cilia: from materials to applications

**DOI:** 10.1038/s41378-023-00611-2

**Published:** 2023-12-13

**Authors:** Seongjin Park, Geonjun Choi, Minsu Kang, Woochan Kim, Jangho Kim, Hoon Eui Jeong

**Affiliations:** 1 Department of Mechanical Engineering, Ulsan National Institute of Science and Technology (UNIST), Ulsan, 44919 Republic of Korea; 2 Department of Convergence Biosystems Engineering, Chonnam National University, Gwangju, 61186 Republic of Korea; 3 Interdisciplinary Program in IT-Bio Convergence System, Chonnam National University, Gwangju, 61186 Republic of Korea

**Keywords:** Nanofabrication and nanopatterning, Nanostructures

## Abstract

Microscale and nanoscale cilia are ubiquitous in natural systems where they serve diverse biological functions. Bioinspired artificial magnetic cilia have emerged as a highly promising technology with vast potential applications, ranging from soft robotics to highly precise sensors. In this review, we comprehensively discuss the roles of cilia in nature and the various types of magnetic particles utilized in magnetic cilia; additionally, we explore the top-down and bottom-up fabrication techniques employed for their production. Furthermore, we examine the various applications of magnetic cilia, including their use in soft robotics, droplet and particle control systems, fluidics, optical devices, and sensors. Finally, we present our conclusions and the future outlook for magnetic cilia research and development, including the challenges that need to be overcome and the potential for further integration with emerging technologies.

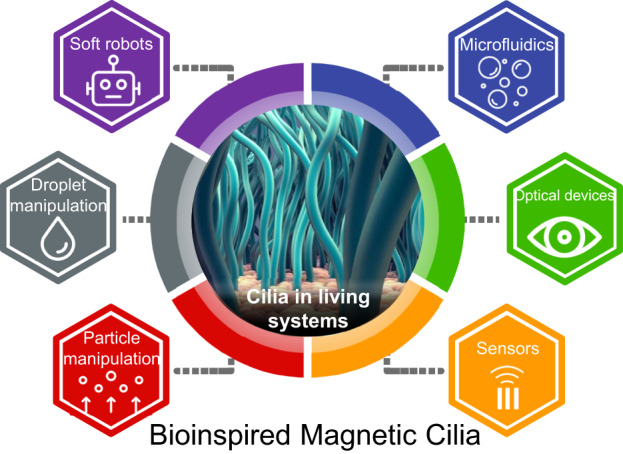

## Introduction

**Fig. 1:**
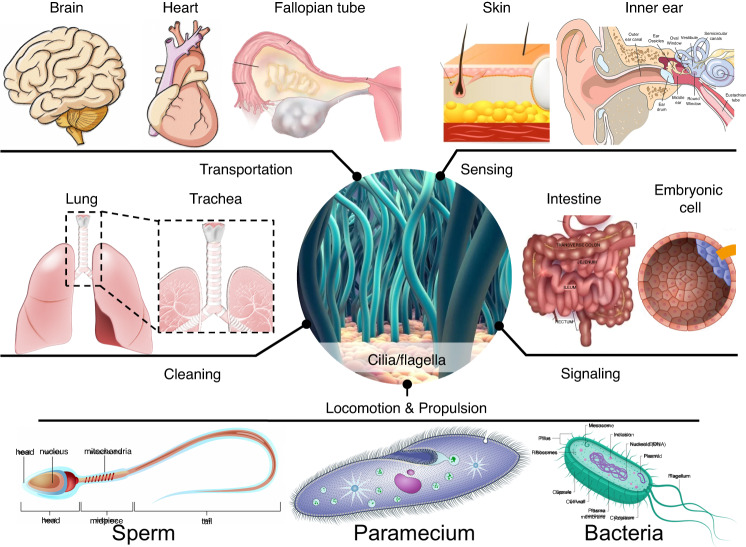
Cilia and flagella found in various living systems and their functions

Cilia are slender appendages adorned with hair-like structures that are present in numerous living organisms. These elegant structures possess precise ultrastructural features, with nanoscale diameters (20–150 nm) and microscale lengths (5–50 µm), and they are constructed from intricate protein arrangements^1^. Remarkably, these tiny cilia utilize molecular motor proteins to generate a range of dynamic beating motions. Despite their minuscule nanoscale dimensions, cilia play diverse and essential biological roles in various living organisms due to their exquisite ultrastructures and self-organized actuating properties (Fig. 1). For example, the cilia carpet present on epithelial cells in the brain^2–4^, heart^5,6^, and fallopian tubes^1,7,8^ facilitates the transportation of biological fluids, while that in the skin and inner ear enhances sensing capabilities^9,10^. Additionally, motile cilia in the airways, such as the lungs and trachea, contribute to the removal of foreign particles, serving a cleaning function^11–13^. Furthermore, cilia observed in the intestine and embryonic cells contribute to tissue growth by mediating diverse forms of extracellular communication through ciliary signaling pathways^14–16^. Moreover, motile cilia and flagella found in microorganisms enable efficient microscale locomotion and propulsion^17,18^.

Inspired by the dynamic self-beating ciliary architectural systems found in nature, a wide range of artificial cilia exhibiting dynamic shape morphing and actuation abilities driven by various mechanisms (e.g., electricity^19,20^, heat^21,22^, light^23,24^, and magnetism^25–27^) have been developed for diverse applications, such as wetting control^28–30^, liquid mixing^31,32^, and actuation^33,34^. Among these artificial cilia, magnetic field-responsive synthetic cilia have recently garnered significant attention due to their unique advantages, such as their remote wireless controllability, immediate field responsiveness, facile field control (e.g., direction and strength), and contactless and nondestructive magnetic fields. The notable advantages of artificial magnetic cilia have expanded their potential beyond simple actuation demonstrations to encompass a broad range of applications, including bioinspired metachronal actuation^8,35^, active droplet manipulation^36–38^, drop bouncing control^39,40^, microfluidics^35,41,42^, antifouling^26,43^, anti-icing^39,44^, energy harvesting^45–47^, light transmittance modulation^48–53^, adhesion control^54,55^, particle manipulation^37,56^^,57^, sensing^58–62^, and soft robotics^63–66^. Therefore, it is important to date to provide an overview of the latest research on magneto-responsive artificial cilia and discuss challenges and future directions for next-generation ciliary devices and systems. This review is divided into three main sections. In the first section (Chapter 2), we introduce the key magnetic components of magneto-responsive artificial cilia and discuss both top-down and bottom-up fabrication strategies, including their material aspects. In the second section (Chapter 3), we explore various applications of magneto-responsive artificial cilia. Finally, we examine the limitations of recent approaches and outline future directions for developing advanced dynamic systems based on artificial magnetic cilia (Chapter 4).

## Materials, design, and fabrication of magneto-responsive artificial cilia

Natural cilia possess long, hair-like ultrastructures with exceptional aspect ratios (ARs) that exceed those achievable with typical fabrication techniques. To develop artificial cilia structures that can closely mimic high-AR natural cilia at the microscale or nanoscale, accurate fabrication strategies are needed. In addition, proper selection of magnetic materials is necessary to give the cilia desirable field-responsiveness when serving as sources of dynamic actuation. Two primary strategies have been employed to create magneto-responsive artificial cilia—top-down and bottom-up approaches—each with their own advantages and limitations. In this section, we first discuss the key magnetic components of artificial magnetic cilia. We then present recently reported strategies for fabricating magnetically actuated artificial cilia, with a particular focus on the fabrication process and specific materials used for the cilia.

### Magnetic components and actuation mechanisms

**Fig. 2:**
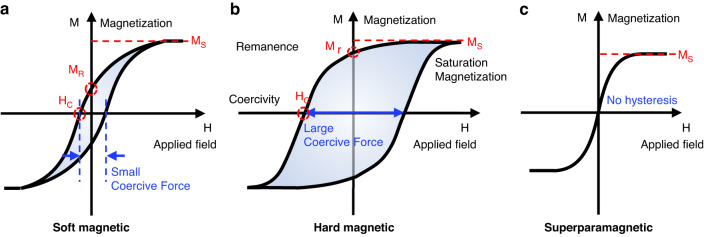
Classification of magnetic materials based on their magnetic behaviors. Hysteresis loop of (**a**) soft magnetic, (**b**) hard magnetic, and (**c**) superparamagnetic materials

The development of artificial cilia has primarily involved a composite mixture of polymer and magnetic particles^67–69^. Biomimetic magnetic cilia have also been created through the colloidal assembly of magnetic particles without a polymeric matrix^70–72^. In both cases, magnetic materials, typically in the form of particles, play a crucial role in enabling dynamic field-responsiveness. These particles can have various shapes that range from isotropic spheres to anisotropic wires, rods, tubes, or plates and sizes that span from nanoscale to microscale dimensions. Among magnetic components, ferromagnetic particles are the most widely employed in constructing magnetic cilia due to their robust response to external magnetic fields. Ferromagnetic particles are microscopic entities made of materials that exhibit ferromagnetism, which is a unique property allowing them to retain their magnetization even after the removal of an external magnetic field. This characteristic arises from the alignment of magnetic domains within the material, resulting in a net magnetic moment. Typically, ferromagnetic particles consist of iron, cobalt, nickel, and their alloys. Hysteresis describes the tendency of a ferromagnetic material to retain some magnetization even when the external magnetic field is removed due to the energy required to reorient the magnetic domains of the material. The relationship between the applied magnetic field and the resulting magnetization of a ferromagnetic material is nonlinear and represented by a hysteresis loop (Fig. 2).

Coercivity, remanence, saturation magnetization, and susceptibility are important properties of ferromagnetic particles (Fig. 2a). Coercivity (*H*_*c*_) refers to the strength of the opposing magnetic field required to remove the magnetic field of the ferromagnetic particle, and it is a measure of a material’s resistance to demagnetization. Remanence (*M*_*r*_) is the magnetic moment that remains in a material after the external magnetic field is removed. Saturation magnetization (*M*_*s*_) is the maximum magnetic moment that can be achieved in a magnetic material when it is fully magnetized. Susceptibility (*χ*) is the degree to which a material is magnetized in response to an applied magnetic field.

Ferromagnetic particles can be classified as soft or hard based on their magnetic properties. Soft magnetic particles exhibit low coercivity and low remanence, facilitating magnetization and demagnetization (Fig. 2b). These materials have low hysteresis losses, making them useful for applications where the magnetic field is frequently changing. Examples of soft magnetic materials include carbonyl iron (CI), iron–silicon alloys, iron–nickel alloys, and iron–cobalt alloys (Table 1). Hard magnetic particles exhibit high coercivity and high remanence, requiring a high magnetic field for demagnetization (Fig. 2a). These materials have high hysteresis losses, making them useful for applications where a permanent magnet is needed. Examples of hard magnetic materials include alnico alloys, samarium–cobalt, hard ferrite, and neodymium–iron–boron (NdFeB) (Table 1).

**Table 1:** Classification of magnetic materials for artificial magnetic cilia

Types of magnetic materials		Characteristics	Examples
Ferromagnetic	Soft magnetic	Low coercivity; low remanence; easy magnetization and demagnetization; small hysteresis loss	Carbonyl iron (CI); iron–silicon alloys; iron–nickel alloys; iron–cobalt alloys
	Hard magnetic	High coercivity; high remanence; strong magnetic field required for demagnetization; large hysteresis loss	Alnico alloys; samarium–cobalt; hard ferrite; neodymium–iron–boron (NdFeB)
Superparamagnetic		Ferromagnetic or ferrimagnetic particles smaller than a few nanometers (typically 1–30 nm); zero coercivity; zero remanence; lower saturation magnetization and higher susceptibility than ferromagnetic particles	Iron oxide nanoparticles (γ-Fe_2_O_3_, α-Fe_2_O_3_, Fe_3_O_4_); cobalt nanoparticles; nickel nanoparticles

Superparamagnetic particles have been widely used to construct artificial magnetic cilia. Superparamagnetism occurs when the size of a ferromagnetic or ferrimagnetic particle is reduced to at most a few nanometers. At these small sizes, thermal energy can overcome the energy barriers that maintain the magnetic moment of the particle, causing the magnetic moment to fluctuate randomly. This finding suggests that superparamagnetic particles do not exhibit permanent magnetization in the absence of an external magnetic field. Instead, the particles exhibit magnetization only when an external magnetic field is applied. The critical size below which superparamagnetism occurs depends on the material and the strength of the magnetic anisotropy, but it is typically in the range of 1–30 nm. As the particle size increases beyond this critical size, the thermal energy becomes insufficient to overcome the energy barriers, and the particle exhibits increasing magnetization. Accordingly, the particles have zero remanence and zero coercivity (Fig. 2c). Superparamagnetic particles have a lower saturation magnetization than ferromagnetic particles due to their small sizes. Instead, superparamagnetic particles are more susceptible than ferromagnetic particles due to their ability to rapidly magnetize and demagnetize in response to changes in the magnetic field. Some examples of materials that exhibit superparamagnetism include iron oxide nanoparticles, cobalt nanoparticles, and nickel nanoparticles (Table 1).

Artificial magnetic cilia incorporating these magnetic particles can manifest dynamic actuation when subjected to either a uniform or nonuniform magnetic field. In a uniform magnetic field, the embedded magnetic particles within the magnetic cilia align themselves in parallel with the field lines. This alignment leads to torques acting on the cilia, subsequently causing bending in a direction determined by the magnetic field orientation^63,73,74^. Similarly, for a nonuniform magnetic field gradient, torque-driven bending actuation occurs. However, within a nonuniform field, magnetic particles within the cilia encounter varying magnetic forces due to the gradient. These differential forces collectively generate net forces that contribute to the bending of the magnetic cilia^75–77^.

### Top-down fabrication strategy for artificial magnetic cilia

**Fig. 3:**
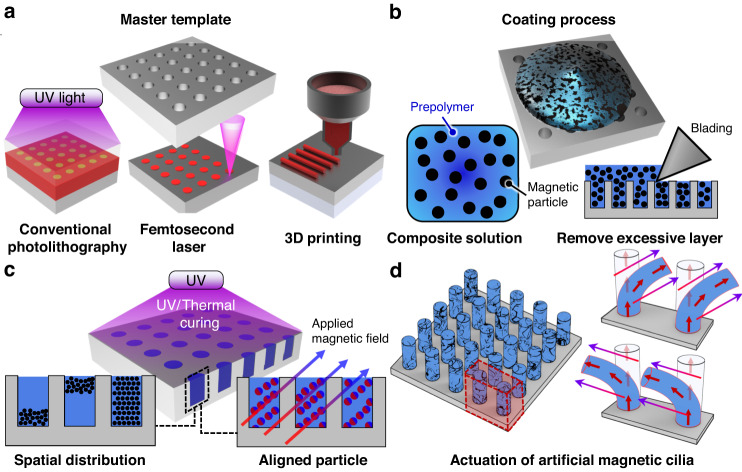
Top-down fabrication of magnetic cilia. **a** Preparation of the master template using various fabrication techniques. **b** Coating process in which polymer composites composed of a prepolymer and magnetic particles are molded onto the prepared master template. **c** Curing of the molded polymer composites through UV/thermal exposure. The spatial distribution or alignment of magnetic particles within the composites can be controlled during the curing process. **d** Resulting cilia obtained after the demolding process, along with their field-responsive actuations

Top-down fabrication is a reliable and straightforward method for producing magnetic cilia with well-defined geometries. Among the various types of top-down techniques, the replica molding technique is widely used for generating artificial magnetic cilia. This method involves replicating a template with negative hole arrays using a mixed solution of precured polymers and magnetic particles (Fig. 3). Specifically, a master template or mold is prepared using various microfabrication techniques (Fig. 3a). Typically, templates are made by photolithography using certain photoresists, such as SU-8, because the photolithographic process can reliably produce microhole array patterns with well-controlled geometry^35^^,56,68,78–81^. Patterned photoresist, Si wafer, or polydimethylsiloxane (PDMS)-based templates can be successfully prepared by the photolithographic process. One of the main limitations of conventional photolithography is the low pattern resolution, which is limited to microscale features. Accordingly, a template with nanoscale holes cannot be easily obtained with conventional photolithography^35^^,57^^,82–84^.

To address this limitation, Luo et al. utilized interference lithography^48^^,67^. The scholars demonstrated that high-density submicron hole arrays with 600-nm diameter and 4–7-µm depth can be prepared by Lloyd’s mirror interference lithography with a 325-nm laser. Unlike conventional photolithography, which requires a photomask, interference lithography enables the direct formation of nanoscale patterns over a large area without the need for a photomask. However, it is difficult to pattern sub-100-nm features even with interference lithography. The femtosecond laser technique^82,85^ can also be used for the direct pattern generation of a master template without a photomask. In recent years, 3D printing has been increasingly utilized in template preparation^8,86–88^. A template mold with varied hole depths and cross-sections can be fabricated simply and inexpensively without complicated photolithographic processes or expensive equipment. However, a drawback of the 3D printing approach is that the pattern resolution is typically limited to hundreds or tens of microns. Instead of fabricating a template mold, commercialized membranes, such as polycarbonate track-etched (PCTE) options, have been used as template molds^89–92^. PCTE membranes with varied pore sizes are available on the market. However, the random pore distribution of the PCTE membrane is a limitation.

Once the template is prepared by the aforementioned methods, the next step is the coating of the template with a composite solution (ferrofluid) of magnetic particles and a prepolymer (Fig. 3b). The magnetic particles within the composite solution are crucial in determining the magneto-responsive properties of synthetic cilia, as discussed in Chapter 2.1. A variety of magnetic particles with different compositions, magnetic properties, and sizes have been used in the preparation of the mixed solution. For example, iron oxide nanoparticles, carbonyl iron microparticles, Co nanoparticles, Co nanowires, NdFeB microparticles, strontium–ferrite microparticles, and electroplated Ni particles have been utilized as the main magnetic components of ferrofluid (Table 2). Superparamagnetic iron oxide nanoparticles are one of the most widely used magnetic materials with sizes ranging from 5 to 30 nm, and they are classified in three different types: maghemite (γ-Fe_2_O_3_), hematite (α-Fe_2_O_3_), and magnetite (Fe_3_O_4_). Ferromagnetic CI particles are also heavily used as magnetic materials with sizes ranging from 1 to 50 µm. CI particles with microscale diameters have higher magnetization values than magnetic nanoparticles and can induce sufficient bending responses under a magnetic field. However, the microscale diameters of the CI particles are only suitable for preparing synthetic cilia with microscale diameters. Artificial nanocilia with small diameters comparable to those of biological cilia cannot be generated using CI particles^92^. Iron oxide nanoparticles with diameters ranging from 5 to 30 nm are suitable for the fabrication of nanoscale artificial cilia. However, iron oxide nanoparticles are vulnerable to particle agglomeration, limiting both the maximum loading concentration of the particles in the solution and the bending responses of the magnetic cilia^67,92^.

**Table 2:** Variations in materials, sizes, and magnetic properties observed in various artificial magnetic cilia and their fabrication and applications

Magnetic particle	Magnetic property	Particle size	Polymer matrix	Length of cilia (AR)	Fabrication	Applications	Refs.
Maghemite (γ-Fe_2_O_3_)	Superparamagnetic	200 nm	PDMS	25 µm (<25)	Molding	N/A	^90^
		900 nm	Polyethylene glycol (PEG)	25 µm (<3)	Self-assembly	N/A	^118^
Magnetite (Fe_3_O_4_)	Superparamagnetic	7−10 nm	PDMS	6.6 µm (<11)	Molding	Optical devices, particle manipulation, microfluidics	^48,67,95^
		13 nm	N/A	N/A	Self-assembly	N/A	^107^
Silica coated magnetite (Fe_3_O_4_/SiO_2_)	Superparamagnetic	30 nm	Polyurethane acrylate (PUA)	40 µm (<8)	Molding	Droplet and particle manipulation	^56,80^
Carbonyl iron (Fe)	Ferromagnetic	70 nm	PDMS	300 µm (<3)	Molding	Microfluidics	^96^
		2.7 µm	PDMS	60 µm (<2)	Molding	Optical devices	^28^
		4.36 µm	Urethane acrylate	3 mm (<15)	3D printing	N/A	^87,106^
		5 µm	PDMS	350 µm (<7)	Molding	Microfluidics, droplet and particle manipulation, soft robotics	^35,57,82,83,86^
		5−50 µm	PDMS	1.6 mm (<11)	Self-assembly	Droplet manipulation	^44^
		2 µm	N/A	50 µm (<25)	Self-assembly	N/A	^124^
Cobalt (Co)	Ferromagnetic	2 µm	PDMS	3.5 mm (<3.9)	Molding	Particle manipulation	^69^
		19.4 nm	PCEMA-b-PAA	N/A	Self-assembly	N/A	^108^
NdFeB	Ferromagnetic	5 µm	PDMS	46 µm (<2.7)	Molding	Droplet manipulation	^68^
			Ecoflex	4 mm (<5)	Molding	Microfluidics, soft robotics	^8,31^
Strontium–ferrite (SrFe_12_O_19_)	Ferrimagnetic	1.4 µm	Urethane acrylate	8 mm (<5.3)	3D printing	N/A	^87^
Nickel (Ni)	Ferromagnetic	11.5 nm	N/A	N/A	Self-assembly	N/A	^126^
Magnetite (Fe_3_O_4_)	Ferrimagnetic	130–373 nm	N/A	10 µm (<27)	Self-assembly	N/A	^99^

For the prepolymer of the composite solution that forms a polymeric matrix of magnetic cilia, precured polymers of PDMS, PDMS-based copolymers, Ecoflex, and polyurethane acrylate (PUA) have been utilized (Table 2). Among these materials, PDMS prepolymer has been the most frequently used due to its soft and tunable mechanical properties and its easy processability. The composite solution made of prepolymer and magnetic particles typically has high viscosity, which limits the filling of the solution into the cavity of the master template. In this case, the viscosity of the composite solution can be properly adjusted by adding certain solvents, such as toluene and hexane, into the solution. An external magnetic field can be applied to the composite solution coated over the template to better fill the cavity of the template during the coating process.

After coating the ferrofluid, excessive residues can be removed from the template surface by mechanical blading, and the template surface is coated with pure elastomers. Then, the template filled with ferrofluid was thermally or UV cured (Fig. 3c). During the curing process, the magnetization direction of the magnetic particles inside each cilium can be aligned in a specific direction by controlling the direction of the external magnetic field^8,28,83,87,93^. The spatial distribution of magnetic particles inside the cilia can also be accurately controlled by adjusting the external magnetic field, enabling the generation of magnetic cilia with nonhomogeneous particle distributions^56,80^. By controlling the magnetization directions and spatial distributions of magnetic particles (i.e., magnetic anisotropy), the magnetomechanical response of the cilia, such as the bending direction and degree, can be programmed into the cilia array. For instance, magnetic cilia arrays with magnetic particles with controlled magnetization directions and spatial distributions can generate metachronal waves with programmable phases, while cilia arrays with magnetic particles featuring uniform magnetization directions and spatial distributions can only generate simple synchronous waves across the entire array^8,35,56,80^.

Finally, the cured cilia are removed from the template mold, resulting in artificial magnetic cilia. The resulting cilia demonstrate instantons and reverse actuating motions under a remote magnetic field^33,84,90,94^ (Fig. 3d). However, the resulting cilia tend to show lateral collapse (mating with adjacent cilia) or bottom collapse (stick to the bottom surface) during the demolding process due to their high AR and low elastic modulus values^25,67^^,92^; these parameters limit the top-down replica molding approach.

In most previous studies on magnetic cilia, the scholars used top-down replica molding and soft lithography techniques due to their simplicity and ability to create artificial cilia with well-defined, ordered geometry^35^^,56^^,68,78–80^. However, these techniques are only suitable for generating vertically aligned cilia arrays. For horizontally aligned, freestanding, flap-type magnetic cilia, photolithographic or 3D printing processes using photopolymerizable resins with embedded magnetic particles have been utilized^95–98^. Belardi et al. used a two-color lithography process to fabricate a freestanding, flap-type magnetic cilia array using photopolymerizable resins with embedded magnetic particles^95^. The composite consisted of a photoreactive copolymer, n-butylacrylate and methacryloyloxybenzophenone, along with magnetite nanoparticles with diameters of 10 nm. To create the cilia, a hydrophilic polymer layer was deposited and crosslinked using UV radiation, and a sacrificial layer was patterned using 2-oxypropylacrylate. The magnetic composite was then deposited and structured into the artificial cilia using UV light, and the sacrificial layer was subsequently removed to release the cilia. Anchoring strips were used to connect one end of each cilium to the surface of the substrate, allowing the remaining parts of the cilia to move freely in response to an external magnetic field.

**Fig. 4:**
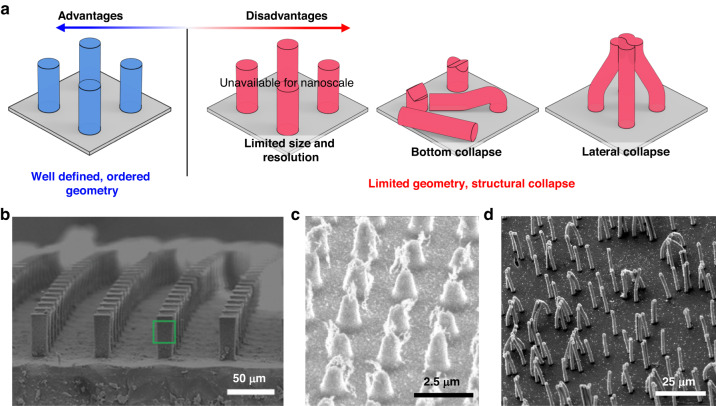
Advantages and disadvantages of the top-down fabrication approach for artificial magnetic cilia. **a** Schematic illustration depicting the advantages and disadvantages of the top-down fabrication method for magnetic cilia. **b** SEM image displaying a well-defined and ordered magnetic cilia array produced by top-down fabrication. Reproduced with permission from ref. ^68^. Copyright 2018, John Wiley and Sons. **c** SEM image showing a magnetic cilia array with limited AR prepared using the top-down process. Reproduced with permission from ref. ^48^. Copyright 2019, American Chemical Society. **d** SEM image demonstrating the structural collapse of the top-down fabricated magnetic cilia array. Reproduced with permission from ref. ^92^. Copyright 2021, National Academy of Sciences (CC BY-NC-ND 4.0)

The aforementioned top-down techniques enable the fabrication of magnetic cilia arrays with precisely defined geometries, such as diameter, height, spacing, and position (Fig. 4a, b)^68^. However, the limited resolution and structural collapse during mold removal are the major limitations of the top-down approach (Table 3). Artificial cilia with nanoscale diameters and exceptional ARs comparable to those of natural cilia are not easily accessible with top-down approaches (Fig. 4a–d)^48,92^. Additionally, these cilia require predefined templates and sophisticated multistep lithographic processes. Interestingly, biological cilia with nanoscale diameters and microscale lengths are formed via a self-assembly process with ciliary building blocks^99–101^.

**Table 3:** Fabrication strategies for artificial magnetic cilia: Advantages and disadvantages

Type	Fabrication techniques	Advantages	Disadvantages
Top-down	Replica molding (template prepared by photolithography)	Well-defined geometries; scalability	Low resolution (micron); photomask needed; low aspect ratio
	Replica molding (template prepared by interference lithography)	Well-defined geometries; high resolution (submicron); maskless lithography	Difficult to pattern sub-100 nm features; low aspect ratio; limited scalability
	Replica molding (template prepared by 3D printing)	Well-defined geometries; simple process; low cost	Low resolution (tens of microns); low aspect ratio
	Direct 3D printing	Multimaterial printing; fast and simple process	Low resolution
	Two-color lithography	High resolution	Complex process
Bottom-up	Self-assembly of ferrofluid	Simple process; high aspect ratio; large area fabrication; template-free	Random spatial distribution; limited control over geometry; low resolution (tens of microns)
	Liquid-phase self-assembly of magnetic particles	High resolution (nanoscale)	Additional binding process required (depending on the magnetic property); low aspect ratio and difficult to fabricate vertical 3D structure (due to capillary forces)
	Vapor-phase self-assembly of magnetic particles	High resolution (nanoscale); high aspect ratio	Additional binding process required (depending on the magnetic property); limited scalability

### Bottom-up fabrication strategy for artificial magnetic cilia

**Fig. 5:**
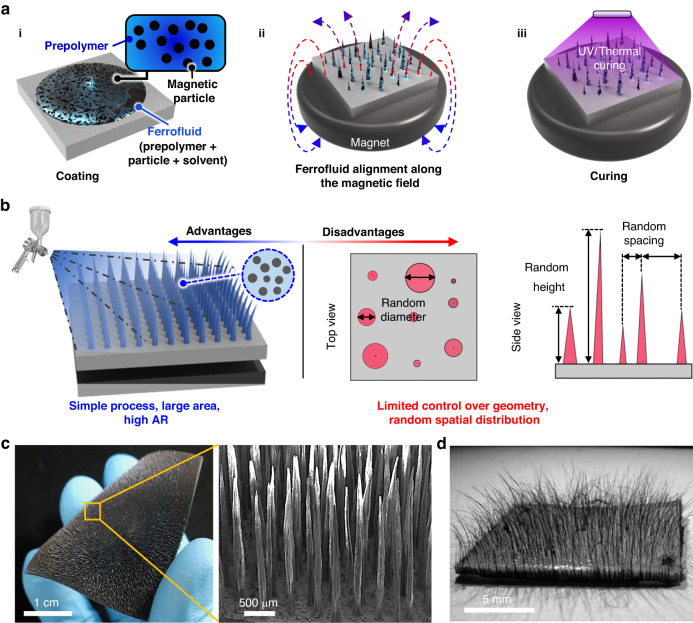
Advantages and disadvantages of the bottom-up fabrication approach for artificial magnetic cilia. **a-i** Coating process involving the placement of a ferrofluid composed of a prepolymer and magnetic particles onto the substrate. **a-ii** Alignment of the ferrofluid along a magnetic field by positioning the substrate in the center of a permanent magnet. **a-iii** Curing of the aligned ferrofluid through UV/thermal exposure, resulting in the formation of the magnetic cilia structure. Schematic illustration depicting the advantages and disadvantages of the bottom-up fabrication method for magnetic cilia. **b** Schematic illustration depicting the advantages and disadvantages of the bottom-up fabrication method. **c** SEM image showing a high-AR magnetic cilia array fabricated using the bottom-up process. Reproduced with permission from ref. ^39^. Copyright 2018, American Chemical Society. **d** Photograph showing the random spatial distribution of a bottom-up fabricated magnetic cilia array. Reproduced with permission from ref. ^105^. Copyright 2010, American Chemical Society

A series of studies have reported that artificial magnetic cilia can also be prepared through template-free self-assembly techniques using a composite solution of prepolymer and magnetic particles (ferrofluid)^29,102–105^. Figure 5a shows a schematic of the template-free approach for self-assembled magnetic cilia based on a ferrofluid. First, a mixed solution of magnetic particles and prepolymers is coated over a substrate that is placed on a permanent magnet (Fig. 5a–i). Additional solvents, such as hexane and toluene, can be added to the mixture to adjust the viscosity of the solution. Then, the mixture is spontaneously aligned along the magnetic field direction and forms conical cilia structures (Fig. 5a-ii). Subsequent thermal or photocuring of the mixed solution results in an elastic magnetic cilia array (Fig. 5a-iii). The resulting cilia can exhibit dynamic actuating behavior by modulating the external magnetic field due to the embedded magnetic particles. Different types of magnetic particles can be used for magnetic particles, which include CI microparticles^28,35,44,57,82,83,86,87,96,106^, iron oxide microparticles or nanoparticles^48,56,67,80,95,107^, Co nanoparticles^69,108^, NdFeB microparticles^8,31,68^, and magnetic beads comprising polystyrene microparticles and embedded magnetic nanoparticles^101^. For the matrix polymer, PDMS is the most commonly used^30,39,64^, but other elastomeric polymers, such as EcoFlex^8,31,63,109^ and poly (styrene-block-isoprene-block-styrene)^105^, and shape memory polymers^110^ can be used.

The greatest advantage of this self-assembly strategy based on ferrofluids is its simple process without requirements for any predefined mask, mold, and template, but it has complicated and time-consuming cleanroom processes (Fig. 5b, Table 3). A magnetic composite solution under a magnetic field gradient can be simply transformed to a linear chain of magnetic particles and subsequent high-AR artificial cilia over a large area by a subsequent curing process (Fig. 5c, d)^39^. Accordingly, many scholars have harnessed this strategy for diverse applications^49,58,111–113^. However, this template-free approach that utilizes ferrofluids typically results in cilia arrays with random spatial distribution (Fig. 5c, d)^105^. Accurate control of the position of individual cilia and intercilia distance is not achievable with this approach. It is also challenging to precisely control the diameter and height of the cilia. Additionally, this technique cannot generate nanoscale cilia. The resulting cilia typically exhibit diameters in the range of a few tens of micrometers and heights in the range of a few hundreds of micrometers or millimeters.

**Fig. 6:**
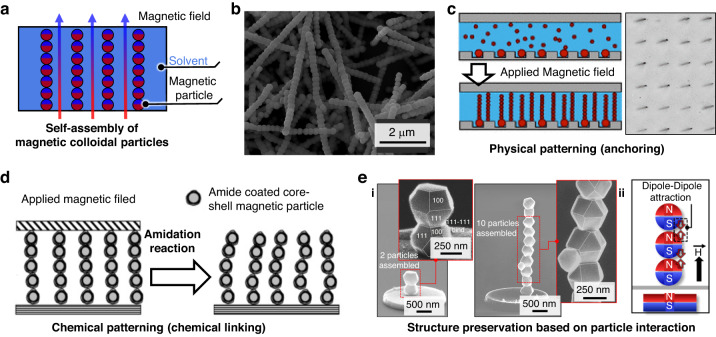
Self-assembly of colloidal magnetic particles without a prepolymer. **a** Schematic illustrating the self-assembly of magnetic colloidal particles under the influence of an applied magnetic field. **b** SEM image displaying the assembled magnetic colloidal particles. Reproduced with permission from ref. ^115^. Copyright 2013, Elsevier. **c** Schematic and optical images presenting a physically patterned colloidal cilia array. Reproduced with permission from ref. ^117^. Copyright 2011, American Physical Society. **d** Schematic image showing a chemically patterned colloidal cilia array. Reproduced with permission from ref. ^118^. Copyright 2005, American Chemical Society. **e** SEM image and schematic image showing the preservation of nanoscale ciliary structures through dipole‒dipole interactions of ferromagnetic or ferrimagnetic particles. **e-i** Reproduced with permission from ref. ^99^. Copyright 2022, John Wiley and Sons. **e-ii** Reproduced with permission from ref. ^124^. Copyright 2022, American Chemical Society

**Fig. 7:**
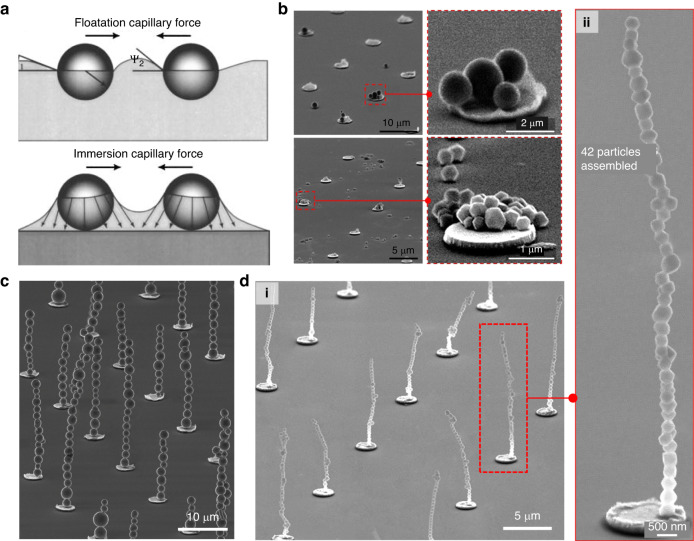
Self-assembly of colloidal magnetic particles without a prepolymer. **a** Schematic illustrating the capillary forces between magnetic particles (top) and between magnetic particles and the substrate (bottom). Reproduced with permission from ref. ^125^. Copyright 2014, American Vacuum Society. **b** SEM images showing the limited geometry achieved through liquid-phase self-assembly. SEM images of the resulting (**c**) microcilia and (**d**) nanocilia fabricated by vapor-phase self-assembly. **b**, **c** Reproduced with permission from ref. ^124^. Copyright 2022, American Chemical Society. **d** Reproduced with permission from ref. ^99^. Copyright 2022, John Wiley and Sons

The techniques for producing artificial magnetic cilia using ferrofluid or a mixed solution of magnetic particles and prepolymer have inherent limitations in constructing nanoscale cilia that resemble natural cilia with nanoscale diameters. This phenomenon occurs mainly due to the high viscosity and composite composition characteristics of the ferrofluids. The utilization of pure magnetic colloidal particles in self-assembly (Fig. 6a), without the inclusion of prepolymers, holds strong potential for the preparation of synthetic cilia at the nanoscale, which closely resemble the exquisite nanoscale structures observed in biological cilia. In particular, field-induced assembly of monodispersed magnetic nanoparticles in a controlled magnetic field can lead to the production of artificial magnetic nanocilia arrays. For example, Frust et al. reported that monodispersed paramagnetic polystyrene beads containing iron oxide nanoparticles can form linear chains when subjected to a magnetic field due to field-induced dipoles^71^. Goubault et al. also reported that superparamagnetic colloidal particles can be self-assembled into magnetic cilia through the application of an external magnetic field^114^. The scholars utilized monodisperse superparamagnetic particles with a diameter of 700 nm that were suspended in an aqueous solution. Upon exposure to an external magnetic field, magnetic dipole moments were induced in the superparamagnetic colloidal particles, resulting in the formation of stable colloidal chains that functioned as artificial magnetic cilia. The particles were coated with polyacrylic acid (PAA), which facilitated irreversible bonding between particles. Wang et al. also generated artificial magnetic cilia using spherical magnetic beads with a diameter of 2.7 µm (polystyrene beads with imbedded magnetic nanoparticles) (Fig. 7b)^115^. These cilia, however, exhibit random spatial distributions, lacking control over their geometries.

The positioning of colloidal cilia can be regulated by utilizing substrates with physically or chemically patterned arrays. Vilfan et al. demonstrated that superparamagnetic particles with a diameter of 4.4 µm can be organized into colloidal chains within rectangular trenches under an external magnetic field^116^. The mechanism of cilia is similar to that reported by Goubault et al.; however, this study resulted in the generation of colloidal cilia arrays with precise positioning. This phenomenon was accomplished through the creation of rectangular trenches in a photoresist layer on a glass slide, with a depth and width slightly larger (5 µm) than the particle diameter (4.4 µm). As a result, a single chain could be formed in each trench upon sedimentation of the particles. To further stabilize the colloidal cilia, a ferromagnetic nickel dot was formed at one end of each trench, serving as an anchoring point under the external magnetic field. The colloidal chains anchored at the nickel dot were capable of generating nonreciprocal beating motion by modulating the direction of the external magnetic field. It should be noted that while microscale cilia were produced using this colloidal self-assembly method, the use of magnetic nanoparticles instead of magnetic microparticles could potentially yield nanoscale cilia. Coq et al. also successfully generated colloidal magnetic cilia with controlled geometries based on superparamagnetic particles with a diameter of 700 nm by utilizing a soft lithographically replicated PDMS substrate with square hole arrays (width: 6 µm, depth: 3 µm, and pitch: 30 or 40 µm) (Fig. 6c)^117^. The scholars used a single paramagnetic particle of 3.5 µm diameter to anchor the cilia.

Instead of physically patterned substrates, chemically patterned substrates can be used for the formation of magnetic cilia with patterned arrays. Singh et al. prepared a glass surface of a microchannel patterned with amine functionality through microcontact printing^118,119^. Then, carboxylated magnetic beads could be selectively anchored on the patterned amine functionality. The nonadsorbed magnetic beads remaining in suspension formed linear chains upon application of a magnetic field. The core–shell magnetic beads consisted of polystyrene beads coated with polyelectrolyte layers and maghemite nanoparticles and were approximately 800 nm in diameter.

It is noted that magnetic cilia assembled from paramagnetic or superparamagnetic particles can be disassembled when the external magnetic field is removed; therefore, an additional binding process is required for irreversible binding between the particles. A variety of surface chemistries have been proposed for this purpose, including the use of PAA-adsorbed magnetic particles^114^, amine-coated beads linked by glutaraldehyde (Fig. 6d)^118^, streptavidin-coated beads linked with a polyethylene glycol linker bifunctionalized with biotin^120^, DNA-functionalized particles^121–123^, and carboxylated beads^118^. Alternatively, ferromagnetic or ferrimagnetic particles can be used for the irreversible formation of artificial cilia. In this case, the cilia can maintain their chain configuration through magnetic dipole‒dipole interactions^72,99,124^, even in the absence of an external field, regardless of the surface chemistry (Fig. 6e).

The majority of the self-assembly techniques described above for magnetic nanoparticles have usually been carried out in a liquid medium, relying on surface tension and evaporation at the liquid/air interface. Although this method is effective for assembling nanoparticles, the strong capillary forces between magnetic particles and between magnetic particles and the substrate make it challenging to precisely manipulate individual particles at the single-particle level and to assemble magnetic particles into a vertical 3D structure (Fig. 7a, b)^124,125^. Furthermore, it is challenging to transport high-AR magnetic cilia that are synthesized in liquid to the air, as the capillary forces at the liquid‒gas interfaces cause the structures to stick together during the process of evaporation^99,100,124–129^.

The limitations of traditional liquid-phase self-assembly of magnetic nanoparticles can be effectively addressed through the use of vapor-phase magnetic particles^99,100,124,126^. For instance, Kang et al. utilized magnetic particles in the vapor state as building blocks for self-assembly (Fig. 7c, d)^99,124^. The scholars showed that these individual magnetic particles, in the form of aerosols, could be assembled layer-by-layer into long, vertical architectural structures on patterned nickel islands under the influence of an external magnetic field, even at different scales of microparticles and nanoparticles. The resulting nanocilia possessed exceptional 3D structural arrangements, with nanoscale diameters, single nanoparticle resolutions and high Ars. In the presence of a magnetic field, the dipole‒dipole coupling between two neighboring magnetic nanoparticles can be expressed as follows^99,115,130^:

$${F}_{{dd}}=\frac{3{m}^{2}(1-{\cos }^{2}\theta )}{{(s+d)}^{4}}$$

where *θ* denotes the angle between the field direction and the line joining the centers of the particles, while *s* and *d* represent the interparticle distance and the particle diameter, respectively. This equation implies that the magnetic dipole‒dipole interaction is attractive, and therefore, the cilia can maintain their structural stabilities through magnetic dipole‒dipole interactions (Fig. 7c, d), without additional binding agents^99,124^. A notable finding is the selective assembly of magnetic nanoparticles onto patterned nickel island arrays. This phenomenon arises from the concentration and intensification of the magnetic field on the nickel sites when a substrate is placed over a permanent magnet^99,115,124,130–132^.

## Applications of magneto-responsive artificial cilia

The main advantage of magneto-responsive cilia is their ability to be remotely controlled and precisely manipulated using external magnetic fields. This property allows for precise and localized actuation, making magneto-responsive cilia an attractive option for a variety of applications where precise control is critical. Unlike other actuators, magneto-responsive cilia can be designed to be small and flexible, allowing them to be inserted into tight spaces and navigate complex environments. Additionally, magneto-responsive cilia can be made from biocompatible materials, making them suitable for use in medical applications without causing harm to the body. The ability to remotely control and precisely manipulate magneto-responsive cilia has significant implications for a range of fields, including biomedicine, materials science and robotics. In this chapter, we will review several practical applications of artificial magnetic cilia.

### Soft robots

**Fig. 8:**
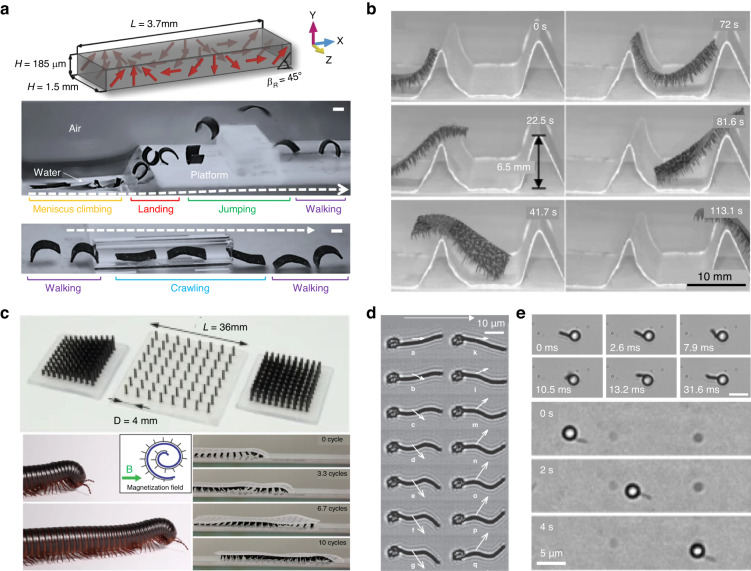
Applications of magnetic cilia in soft robotics. **a** Magnetic soft-bodied robot capable of untethered locomotion through programmed magnetization. Reproduced with permission from ref. ^63^. Copyright 2018, Springer Nature. **b** Untethered soft millirobot featuring multiple tapered soft magnetic feet. Reproduced with permission from ref. ^64^. Copyright 2018, Springer Nature. **c** Soft robots incorporating metachronal magnetic cilia carpet. Reproduced with permission from ref. ^8^. Copyright 2018, Springer Nature. **d** Single-strand magnetic artificial swimmer exhibiting beating motion. Reproduced with permission from ref. ^121^. Copyright 2005, Springer Nature. **e** Single-strand magnetic artificial swimmer demonstrating rotational motion. Reproduced with permission from ref. ^133^. Copyright 2018, Springer Nature

Soft robotics has garnered substantial attention in recent years due to its capacity to adapt to dynamic environments and execute intricate tasks without the need for intricate control algorithms (Fig. 8). One particularly promising avenue within soft robotics research involves the utilization of artificial magnetic cilia as actuators to achieve small-scale, multilegged locomotion. In contrast to prior small-scale robots that exhibit limited mobility in unstructured settings, magneto-responsive macrorobots equipped with dynamic cilia offer remote controllability, flexibility, and heightened degrees of freedom. These characteristics enable them to access and navigate intricate, confined terrains noninvasively (Fig. 8a)^8,63,64,83,93^. Furthermore, these robots can be fabricated from biocompatible materials, positioning them as a propitious tool for biomedical applications, such as minimally invasive surgery, targeted drug delivery, and manipulation of individual cells. Thus, magnetic soft robots are emerging as a promising technology with substantial potential in the realm of soft robotics.

For instance, an untethered soft millirobot featuring a multitapered architecture in its soft feet (cilia) was developed via the self-assembly process described in Chapter 2.3 (Fig. 8b)^64^. This robot was made of a magnetic mixed solution comprising PDMS, hexane, and magnetic iron particles (average diameter: 6–10 μm). The foot (cilia) length and interfoot spacing measured approximately 650 μm and 600 μm, respectively, with a modulus of approximately 2 MPa. Under the influence of a magnetic field, the robot feet underwent tapering and deformation, aligning with the direction of the magnetic flux and facilitating efficient robot movement. Without its specialized foot structure, the robot displayed negligible movement even when exposed to a magnetic field, emphasizing the crucial role of magnetic cilia in soft millirobotics. Intriguingly, the robot demonstrated its ability to operate within challenging environments, including locomotion on wet surfaces coated with a liquid film, propulsion with a load capacity equivalent to 100 times its own weight, and surmounting steep obstacles measuring approximately 10 times its leg length. Furthermore, the robot effectively demonstrated drug transport within a simulated stomach environment characterized by wet conditions.

Numerous cilia in living organisms generate metachronal waves, offering precise control over movement direction during locomotion. By finely adjusting the phase and timing of cilia activation, organisms can navigate and alter their course with exceptional precision. Inspired by these natural ciliary movements, researchers have developed cilia-based soft robots capable of generating metachronal waves (Fig. 8c)^8^. For instance, Gu et al. employed a replica molding technique to create a cilia carpet using a mixed solution of Ecoflex and nonmagnetized NdFeB particles. Subsequently, this cilia carpet was magnetized by wrapping it around a cylindrical template with a predefined curvature and subjecting it to a magnetizing process (Fig. 8c). This procedure resulted in various cilia beating patterns, including metachronal waves. When the resulting cilia carpet, featuring a 4-mm length, 0.8-mm diameter, and 4-mm array periodicity, was inverted and placed on a rigid plastic surface beneath a rotating magnetic field, it functioned as a soft cilia robot, generating a distinctive locomotion pattern. Indeed, the authors designed a soft robot inspired by the locomotion of an African millipede, arranging its legs in metachronal waves (Fig. 8c).

In addition to cilia arrays or carpets, single-strand magnetic cilia have found utility in robotic applications. For instance, microscopic artificial swimmers that closely resemble bacteria with flagella have been developed using a linear chain of colloidal magnetic particles (Fig. 8d)^121^. The researchers constructed a filament by linking a 1-µm superparamagnetic colloid with 107-nm-long (315 base pairs) double-stranded DNAs. Additionally, the researchers controlled the flexibility of the filament by adjusting the length and number of DNA linkers and the particle diameter. Subsequently, these filaments were affixed to red blood cells. The superparamagnetic filament demonstrated the ability to align with an external uniform magnetic field, and it could be actuated through an additional oscillating transverse field. Moreover, a microscale artificial swimmer was developed using a hybrid propeller design (Fig. 8e)^133^. This propeller comprised a paramagnetic sphere and a ferromagnetic nickel nanorod that interacted with each other through dipolar forces. Upon the application of an external magnetic field to the propeller, the nanorod initiated rotation, and the proximity of the sphere facilitated the conversion of this rotational motion into directed propulsion. By modulating the amplitude or frequency of the magnetic field, the direction of propulsion could be altered. Various interplays between magnetism, gravity, and hydrodynamics could yield diverse propulsion mechanisms.

### Droplet and particle manipulation

The manipulation of droplets is essential for a variety of applications, including biochemical analysis devices and bioinspired functional surfaces. Passive methods that employ static micro- or nanopatterned surfaces have certain limitations due to their slow and irreversible nature^134–139^. Active techniques, such as electrowetting^134,135^, surface acoustic waves^136,137^, and thermocapillary force^138,139^, provide enhanced control over droplets, but they require predefined electrodes and external power sources, limiting their scalability and applicability. Although some scholars reported positional control of droplets using a magnetic field, they utilized droplets mixed with magnetic nanoparticles, limiting the broad application of the technique^39,140^. Unlike these conventional approaches, recently reported magnetic cilia offer wireless, active, and simple controllability of various pure liquid droplets and solid particles. Additionally, magnetic cilia can be modified chemically and structurally to have hydrophobic or oleophobic properties. This modification allows the magnetic cilia to operate effectively in a wide range of fluid environments, making them suitable for various applications^50,85,112,141^.

**Fig. 9:**
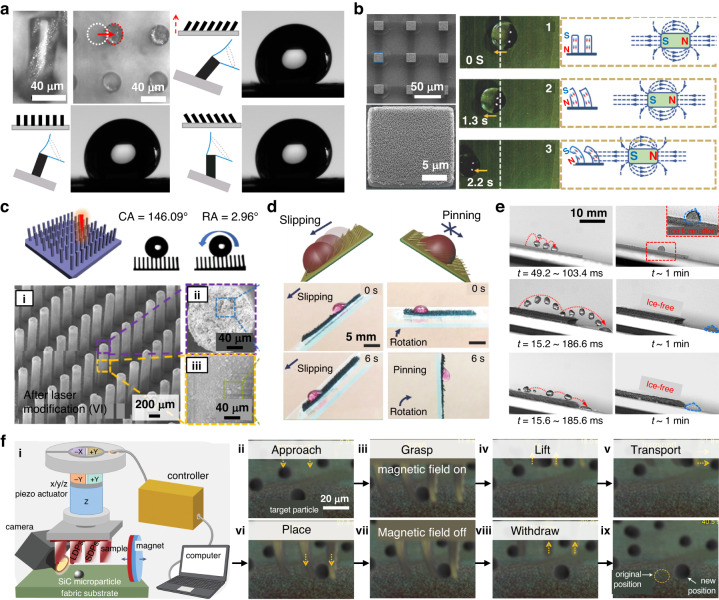
Droplet and particle manipulation processes using magnetic cilia. **a** Active droplet manipulation with a magnetic cilia array. Reproduced with permission from ref. ^142^. Copyright 2014, American Chemical Society. **b** Active droplet manipulation using superhydrophobic magnetic cilia coated with nanoparticles. Reproduced with permission from ref. ^68^. Copyright 2018, John Wiley and Sons. **c** Active droplet manipulation using superhydrophobic magnetic cilia treated with a femtosecond laser. Reproduced with permission from ref. ^82^. Copyright 2020, American Chemical Society. **d** Active droplet manipulation with superhydrophobic magnetic cilia infused with lubricating oils. Reproduced with permission from ref. ^29^. Copyright 2017, John Wiley and Sons. **e** Drop-shedding and anti-icing capabilities of bottom-up fabricated superhydrophobic magnetic cilia. Reproduced with permission from ref. ^39^. Copyright 2018, American Chemical Society. Copyright 2020, John Wiley and Sons. **f** Particle manipulation with a magnetic cilia platform. Reproduced with permission from ref. ^56^. Copyright 2020, John Wiley and Sons

The primary method for actively manipulating droplets involves the use of a magnetic cilia array composed of a polymer and magnetic particle composite. This array can be organized in either a regular or random pattern. For instance, Zhu et al. employed electroplating to create a regular array of ferromagnetic nickel pillars on a soft PDMS substrate^84^. These pillars had dimensions of 26–30 µm diameters and 70–75 µm heights, and they were spaced 60 µm apart. The resulting pillar array induced droplets to spread in the direction of the pillar tilt when subjected to an external magnetic field. Similarly, Drotlef et al. developed a magnetic pillar array using PDMS mixed with CI particles through a soft molding technique (Fig. 9a)^142^. These micropillars measured 43 µm in height and 18 µm in diameter and were spaced 22 µm apart. The researchers demonstrated that wetting behavior could be actively controlled using these pillars. However, it is worth noting that the regular pillar arrays in these two studies did not exhibit superhydrophobic characteristics, which limited the achievement of fast, dynamic, and reversible droplet manipulations.

To overcome this limitation, researchers have developed regular magnetic cilia arrays that exhibit superior water repellency by incorporating nanoscale roughness over microciliary structures. Lin, Y. and colleagues achieved superhydrophobic magnetic cilia by coating SiO_2_ nanoparticles with diameters of 250 nm over the cilia using a dip-coating process (Fig. 9b)^68^. The magnetic cilia were created using a composite of PDMS and NdFeB particles through a soft molding technique. After the SiO_2_ nanoparticle coating, the static contact angle (CA) and sliding angle (SA) values of the magnetic pillar array were significantly enhanced to 151° and 4°, respectively, from the noncoated pillar array CA of 134° and SA of 14°. As a result, the pillar array could simultaneously demonstrate dynamic actuation and superhydrophobicity with low adhesion along the direction of pillar tilt. Ben et al. also developed a superhydrophobic magnetic cilia array using a composite of PDMS and cobalt magnetic particles with a diameter of 2 μm^143^. The scholars soaked the as-prepared microcilia array in a solution of SiO_2_ nanoparticles with an average diameter of 14 nm. This coating process resulted in the formation of microscale/nanoscale hierarchical structures over the cilia array, which led to exceptional superhydrophobicity with a static CA over 150°. The superhydrophobic pillars generated local differences in potential energy in the array when bent by a magnetic field. Furthermore, the actuated pillars provided a driving force for the droplet to move along the direction of the bent pillar, allowing a water droplet to move toward the location of the bent pillars in a reversible manner^140^. Thiol-ene magnetic pillars modified with allyl-terminated microparticles and nanoparticles have also been utilized to achieve superhydrophobic magnetic pillar arrays^144^.

Alternative approaches have been developed to create superhydrophobic magnetic cilia arrays without the need for surface coating using SiO_2_ nanoparticles. For example, one method involved the use of a CO_2_ laser to ablate the surface of a magneto-responsive film composed of PDMS and ferromagnetic oxide nanoparticles with a diameter of 100 nm^145^. Additionally, a femtosecond laser could also be employed to generate irregular hierarchical structures on the surfaces of magnetic pillars made from PDMS and carbonyl iron powders (Fig. 9c)^82^. In both cases, these roughened surfaces allowed the pillar arrays to achieve a static CA greater than 150° and an SA of less than 4°, enabling remote and active manipulation of pure liquid droplets. Apart from laser ablation, another method for creating a slippery magnetic cilia array involves impregnating PDMS composite cilia with lubricating oils, such as silicone or perfluorinated oil. This impregnation significantly reduces the contact angle hysteresis (Fig. 9d)^29,36,146^.

In addition to magnetic cilia with a regular array created through top-down fabrication techniques, researchers have explored the use of bottom-up prepared magnetic cilia for actively manipulating nonmagnetic droplets. For instance, Kim et al. employed magnetically responsive cilia characterized by a random spatial distribution to manipulate liquid droplets^140^. The scholars prepared these cilia using a moldless self-assembly technique involving a ferrofluid consisting of precured polymers and magnetic particles on a permanent magnet. Coating the surfaces of the resulting microcilia with carbon nanoparticles yielded stable superhydrophobicity, with a static CA exceeding 150° and an SA less than 10°, irrespective of the actuating angles of the cilia. As a result, the authors successfully demonstrated active, rapid, precise, and reversible control over the positions and movements of pure discrete droplets using only a permanent magnet placed on the cilia surface.

**Fig. 10:**
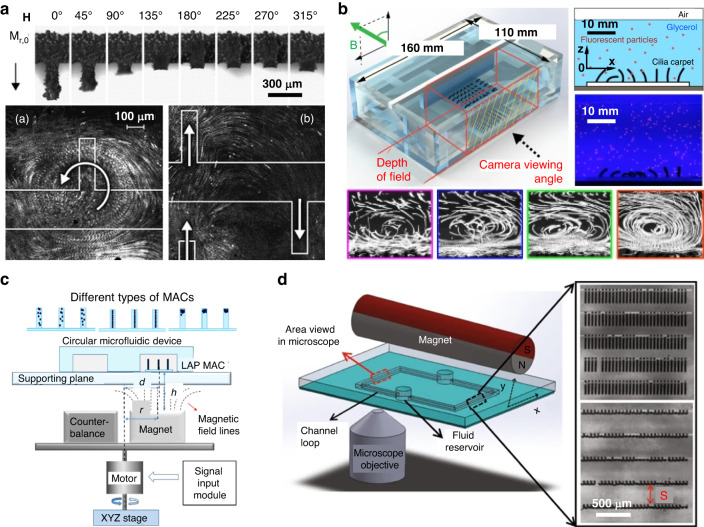
Applications of magnetic cilia in microfluidics. **a** Application of horizontally aligned artificial cilia in microfluidic devices. Reproduced with permission from ref. ^96^. Copyright 2009, Royal Society of Chemistry. **b** Demonstration of enhanced flow rates through metachronal motion generated by artificial magnetic cilia. Reproduced with permission from ref. ^8^. Copyright 2020, Springer Nature. **c** Microfluidic devices integrated with different types of magnetic artificial cilia. Reproduced with permission from ref. ^155^. Copyright 2018, Elsevier. **d** Microfluidic pumping application using artificial magnetic cilia. Reproduced with permission from ref. ^41^. Copyright 2018, Springer Nature

In addition to their horizontal in-plane droplet manipulation capabilities, scholars have recently unveiled the remarkable ability of superhydrophobic magnetic cilia to influence the vertical drop bouncing behaviors on their surfaces^34,39,44,147–150^. These cilia can actively induce specific modes of droplet rebound, including pancake bouncing, directional bouncing, and drop fragmentation. This active control over droplet rebound has the potential to significantly reduce the contact time of falling droplets on the cilia surface, promising practical applications, such as creating drop-shading surfaces and ice-free surfaces (Fig. 10e)^39,44,149,150^. Moreover, these cilia show promise as essential components in open-surface droplet-based microfluidic devices^147^.

In addition to their role in manipulating droplets, magnetic cilia platforms are versatile for transporting and removing solid particles^37,38,56,57^. This functionality closely resembles the motion of motile epithelial cilia that line the inner walls of fallopian tubes and mammalian airways^1^. For instance, Wang et al. demonstrated the directional movement of silicon carbide (SiC) microparticles with a diameter of 50 µm on regular PUA cilia embedded with Fe_3_O_4_@SiO_2_ magnetic nanoparticles (Fig. 9g)^56^. Furthermore, the scholars developed a magnetic microtweezer employing these magnetic PUA cilia, which were connected to a piezoelectric ceramic actuator. By utilizing this system, the authors successfully picked up, transferred, and placed individual SiC particles with an average size of 10 µm, effectively showcasing the practical application of magnetic cilia as microscale tweezers^56^. Beyond particle manipulation, the active manipulation and removal capabilities of magnetic cilia, both for liquid and solid particles, hold the potential for creating self-cleaning and antifouling surfaces. These capabilities can be harnessed by inducing either local or global vortices near the material surface^26,43,151,152^.

### Microfluidics

Microfluidics and lab-on-a-chip devices have garnered significant attention in recent years due to their potential for miniaturization, automation, and high-throughput analysis. However, precise manipulation of fluids and particles in microscale environments remains a challenging task. Traditional microfluidic actuators, such as pneumatic valves and pumps, are burdened by certain limitations, such as complexity, size, and restricted control. Artificial magnetic cilia present a promising solution to these challenges, offering noninvasive, programmable, and reversible actuation capabilities.

Magnetic cilia possess unique attributes that render them ideal for microfluidic applications^8,96,153–157^. First, magnetic cilia provide remote and reversible actuation, enabling precise control over fluid and particle movement within microscale settings. Second, magnetic cilia can be programmed to generate diverse flow patterns, including vortices, spirals, and oscillatory flows, enabling their utilization in the mixing, pumping, and sorting of fluids and particles. Third, the contactless nature of magnetic cilia offers a noninvasive actuation method, mitigating the risks of contamination and damage to biological samples. Last, the flexibility and controllability of magnetic cilia position them as feasible alternatives to conventional microfluidic actuators. By capitalizing on these advantages, artificial magnetic cilia have demonstrated substantial potential for various microfluidic and lab-on-a-chip applications.

A prominent application of magnetic cilia is mixing, where they induce localized vortices or oscillatory flows to facilitate efficient mixing of fluids and particles. In microchannels characterized by low Reynolds numbers, generating turbulence for effective mixing is challenging. Magnetic cilia, particularly those capable of nonreciprocal and metachronic motions, serve as active micromixers to address this issue^154^. Magnetic cilia have proven effective for pumping, generating continuous or pulsatile flow in microfluidic channels. For instance, Fahrni et al. fabricated ferromagnetic artificial cilia utilizing PDMS and iron nanoparticles, achieving rotational and translational fluid movements in an aqueous solution within a microfluidic chamber (Fig. 10a)^96^. These cilia displayed velocities reaching ~0.5 mm s^−1^ through actuation with a homogeneous rotating magnetic field. Furthermore, metachronal waves have been recognized as pivotal mechanisms for facilitating efficient fluid transport within biological systems. Gu et al. conducted an investigation into fluid transport across cilia carpets featuring diverse beating patterns, including synchronous and metachronal waves. Their experimental findings highlighted the ability of metachronal waves to foster the formation of a coherent flow structure, resulting in notably highly effective liquid transport and pumping relative to the synchronous wave (Fig. 10b).^8,153,158^

It is essential to note that precise control over the distribution of magnetic particles within the cilia is a prerequisite for effectively utilizing magnetic cilia as major components in microfluidic devices. For example, Zhang et al. developed magnetic artificial cilia (MAC) through a micromolding process that facilitated the controlled distributions of magnetic particles within the cilia (Fig. 10c)^155^. Three types of MAC were generated: standard with random particle distribution, linearly aligned particle distribution (LAP MAC), and concentrated particle distribution at the cilia tip (CP MAC). Magnetization measurements and cilia bending tests indicated that LAP MAC exhibited superior magnetic properties and actuation performance, while CP MAC exhibited the weakest magnetic response. Consequently, LAP MAC generated substantial flow rates in circular and branched channels, surpassing most previously published artificial cilia and even competing with electrohydrodynamic and electroosmotic pumping methods.

In addition to soft elastomer composite-based cilia, metal- or metal-alloy-based cilia can be harnessed for microfluidic applications. Hanasoge et al. reported microfluidic pumping using artificial magnetic cilia (Fig. 10d)^41^. The scholars fabricated a free-standing magnetic NiFe cilia array using surface micromachining techniques, with each cilium measuring 200 µm in length, 20 µm in width, and 60 nm in thickness and with an intercilia spacing of 20 µm. The cilia array was integrated into a PDMS microchannel. The researchers found that the cilia were indeed effective at pumping fluids within the microfluidic channels. The maximum centerline velocity generated by the magnetic cilia was approximately 1350 μm s^−^^1^, with a volumetric flow rate of approximately 11 μL min^−1^ and a self-pumping frequency of approximately 2.5 min^−^^1^. These results represent the highest reported values for similar ciliary microfluidic pumping systems.

### Optical devices

Optical and photonic devices, which can dynamically and reversibly alter their structures through external magnetic field control, is a field that has witnessed growing interest in recent years. Among these devices, magnetic-cilia-based optical devices stand out due to their remarkable feature of swift switchability achieved solely with the aid of an external magnetic field. This unique characteristic has spurred exploration into various magnetic materials and structures, including magnetic cilia, as they hold great promise for actively manipulating light reflectance or transmittance according to specific requirements.

**Fig. 11:**
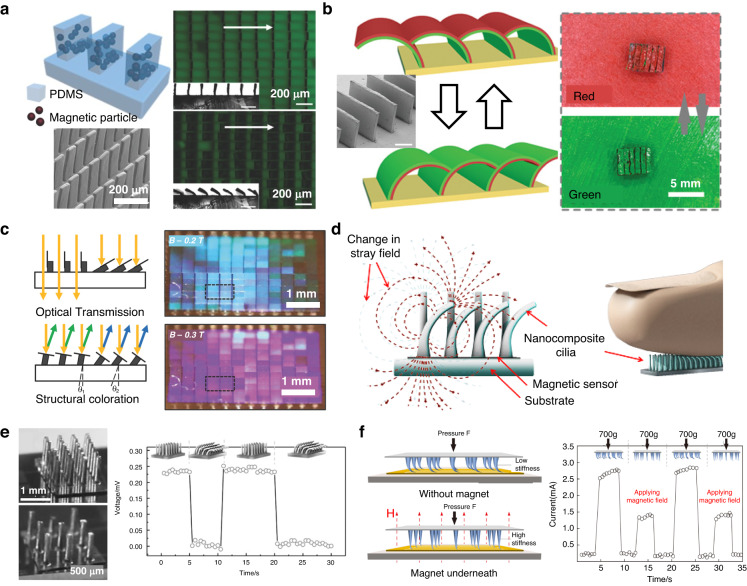
Application of magnetic cilia in optical devices and sensors. **a** Optical switch device based on magnetic plate cilia. Reproduced with permission from ref. ^159^. Copyright 2017, John Wiley and Sons. **b** Dynamic color conversion based on magnetic cilia. Reproduced with permission from ref. ^50^. Copyright 2019, John Wiley and Sons. **c** Tunable optical properties using magnetic microplate arrays. Reproduced with permission from ref. ^51^. Copyright 2017, John Wiley and Sons. **d** Schematic illustrating the working principle of a tactile sensor based on magnetic cilia. Reproduced with permission from ref. ^59^. Copyright 2015, MDPI. **e** Photographs and performance of the tactile sensor based on magnetic cilia. Reproduced with permission from ref. ^60^. Copyright 2015, John Wiley and Sons. **f** Schematic and performance of the pressure sensor based on self-assembled magnetic cilia. Reproduced with permission from ref. ^58^. Copyright 2022, American Chemical Society

A notable instance of magnetic cilia application in optical devices is the development of an optical switch based on magnetic plate cilia (Fig. 11a)^159^. The researchers employed a soft molding technique utilizing a composite solution of PDMS and CI particles to create microplate arrays with magnetic responsiveness. Gold was selectively deposited on one side of the microplate to study the impacts of tilting on reflectivity and fluorescence intensity. A fluorescent dye (coumarin 6) was incorporated into the PDMS substrate. The Au-coated side exhibited relatively high reflectivity due to its high attenuation coefficient, while the PDMS layer on the opposite side, containing CI particles, resulted in relatively low reflectivity due to light absorption and scattering. Switching the microplates between the two sides altered the surface reflectance, transitioning from a relatively high state to a low state. Furthermore, when the microplates were positioned perpendicular to the substrate, fluorescent light could traverse the array interstices, resulting in the highest overall fluorescent intensity. Conversely, tilting the microplates shielded the substrate, leading to a decrease in the overall fluorescence intensity.

Magnetic cilia have also been applied in optics and photonics for the development of dynamic color conversion systems^48,50,51^. Jiang and colleagues proposed a magneto-responsive Janus microplate array to dynamically switch colors (Fig. 11b)^50^. The scholars first fabricated a magnetic microplate array using PDMS and CI powder via soft molding. Then, they applied different colors of red and green on each side of the microplates. By controlling the tilting angle of the array, the surface color could be dynamically switched between red and green, resembling the mechanism by which a chameleon hides itself on a branch. Yang et al. developed a novel method for fabricating responsive surfaces with tunable optical properties (Fig. 11c)^51^. The fabrication process entailed creating an elastomer microcilia array embedded with ferromagnetic nanoparticles on a receiving substrate. This step was followed by producing a silicon scale array on a donor substrate and integrating the prefabricated scales onto the microcilia through transfer printing-based deterministic assembly. The scholars demonstrated that the microcilia exhibited tunable optical properties, including transmittance and structural coloration. The surface for tunable optical transmission was fashioned by affixing bare silicon scales on magnetic micropillars in an out-of-plane configuration, effectively serving as microscale shutters to control light passage. The transmittance of the surface could be continuously adjusted from 30% to 90% in response to an external magnetic field.

### Sensors

Magnetic cilia have emerged as promising tools for developing physical or mechanical sensors that mimic the sensitive mechanosensory hair-like cilia receptors found in nature. These sensors can detect a wide range of external forces, including vibration, fluid flow, and touch. One of the main advantages of magnetic cilia-based sensors is their high sensitivity. When cilia are deflected by an external force, they change the magnetic field detected by a magneto-impedance sensor, providing a simple and efficient method for measuring force (Fig. 11d)^59^.

A tactile sensor was developed by Alfadhel and Kosel, who utilized magnetic nanocomposite cilia prepared using PDMS and iron nanowires (NWs) (Fig. 11e)^60^. Iron NWs were chosen for their biocompatibility and high magnetization at remanence and their high coercivity, making them nanopermanent magnets. Multilayer giant magneto-impedance (GMI) sensors were integrated under the cilia to fabricate tactile sensors. The sensor operated by detecting the change in the cilia magnetic field created by the iron NWs when deflected by an external force, such as vibration, fluid flow, and hand touch. The nanocomposite cilia array sensor with a 9-cilia arrangement could detect vertical forces reaching 50 kPa with a high resolution of 0.23 kPa and a sensitivity of 15–60 mΩ kPa^−1^. The cilia sensor with a 24-cilia arrangement showed a response in a large range reaching 170 kPa with a resolution of 0.88 kPa and a sensitivity of 16 mΩ kPa^−1^. This finding demonstrated the potential of magnetic cilia-based sensors in developing highly sensitive and accurate tactile sensors.

Scholars have previously reported that tactile sensors with unique microstructures, such as micropillars^59^, microdomes^160^, and microwrinkles^161,162^, can achieve better sensing performance than those with planar geometry. However, the fabrication of such microstructures usually involves expensive and complex processes. In contrast, the template-free, self-assembly approach for producing magnetic cilia offers a promising avenue for developing inexpensive and highly sensitive tactile sensors (Fig. 11f)^58^. One such example is the work of Jing et al., who proposed a novel method for creating pressure sensors using self-assembled magnetic cilia coated with carbon nanotubes (CNTs)^162^. Specifically, the researchers first prepared a magnetic cilia array via the self-assembly process of a mixed solution of CI particles and PDMS. The scholars then coated a CNT film onto the cilia, resulting in a piezoresistive ciliary electrode, while a planar composite film coated with CNTs served as the counter electrode. The piezoresistive magnetic cilia and counter electrodes were cut into square shapes and overlapped, with CNT-coated surfaces touching each other. The sensor operated based on the principle of contact mode change between the piezoresistive electrode and the counter electrode, with a transition from point contact to line contact as the pressure increased, leading to an increase in current. The sensor showed impressive pressure-sensing capabilities, with a high sensitivity of 0.497 kPa^−1^ in the low-pressure regime (<100 Pa), a wide sensing range from a few pascals to 80 kPa, and a fast response time of <0.2 s. Lin et al. also reported a pressure sensor utilizing self-assembled magnetic cilia^163^. The scholars successfully measured the relative inductance change between the magnetic cilia and a planar spiral coil upon the application of external pressure. Furthermore, the researchers demonstrated the strong repeatability and durability of the sensor, maintaining their performance integrity even after 5000 cycles.

## Conclusion and perspective

The development of artificial magnetic cilia has opened new possibilities across a wide spectrum of applications, spanning from soft robotics to microfluidics, droplet and particle manipulation, optical devices, and sensors. These artificial cilia closely mimic the motion of natural cilia, demonstrating remarkable precision and efficiency, thus making them a promising technology ripe for a multitude of fields. Recent advancements in magnetic materials, microfabrication techniques, and microscale modeling have allowed for the development of highly sophisticated magnetic cilia systems, enabling researchers to explore previously uncharted territories of study.

However, there are still several limitations that need to be overcome. First, present fabrication techniques struggle to replicate the nanoscale diameter and exceptional AR characteristics of natural cilia. Top-down soft molding employing a composite solution exhibits limited resolution, resulting in magnetic cilia with microscale diameters and constrained AR, primarily due to the high viscosity and low modulus of the composite solution. Additionally, the limited resolution of photolithography used in template molds hinders the creation of nanoscale cilia through the top-down approach. While the bottom-up approach circumvents the need for a predefined template, precise control over cilia geometries, such as position, diameter, and length, remains elusive. Advanced nanofabrication techniques that can generate magnetic cilia with nanoscale diameters, ultrahigh ARs, precise geometry control, and scalability are imperative. In this context, nanoscale 3D printing exhibits the potential to produce such structures; however, it remains relatively expensive and lacks scalability. A synergistic integration of top-down and bottom-up approaches can potentially circumvent this limitation.

Second, it is crucial to ensure structural durability and long-term repeatability under a range of working conditions, including exposure to air, underwater environments, various liquid media, and different temperature conditions. Unlike natural cilia, synthetic counterparts may experience lateral or bottom collapse during transitions between air and underwater conditions, coupled with subsequent drying processes. This issue can be addressed through surface functionalization, aimed at preventing such collapse, in conjunction with the development of highly durable polymeric materials, advanced magnetic components, and additives to provide additional functionalities. Third, magnetic cilia are reliant on external magnetic fields, necessitating the use of bulky permanent or electromagnets. This phenomenon limits their applicability to enclosed, confined spaces and significantly limits their potential uses. If magnetic cilia can be seamlessly integrated with compact, on-board magnetic control systems, their utility can extend to a broad range of practical applications, including macroscale or mesoscale mobile soft robotics.

Overcoming these challenges through further research holds the potential to firmly establish artificial magnetic cilia as indispensable components in various technological domains. For instance, magnetic cilia-based microrobots have the potential to revolutionize drug delivery and surgical procedures, offering improved precision and reduced invasiveness relative to existing methods. The manipulation of droplets and particles through magnetic cilia can drive innovation in microfluidics, ultimately leading to the development of lab-on-a-chip devices for diagnostics and analysis. Expanding upon this basis, magnetic cilia-based functional surfaces equipped with active wetting and droplet rebound control mechanisms have the potential to catalyze the creation of never-wet or never-iced surfaces while simultaneously enabling energy harvesting from falling droplets. Furthermore, magnetic cilia can be employed to induce fluid flows, granting control over the transportation of biological cells or the creation of artificial tissue environments for drug testing. In addition, envisioning applications, such as artificial magnetic cilia for airway stents and highly sensitive hearing aid devices utilizing magnetic cilia, is entirely within the realm of possibility. Moreover, the integration of magnetic cilia with emerging technologies, such as machine learning and artificial intelligence algorithms, can empower them to exhibit autonomous and adaptive behaviors. Within the field of materials science, magnetic cilia may play a pivotal role in the development of smart materials capable of changing shape or altering their mechanical properties in response to external magnetic fields.

Although the field of artificial magnetic cilia remains in its nascent stages, its potential applications and possibilities are vast. Continued research and development efforts hold the promise of uncovering new breakthroughs and advancements, thereby opening novel realms of research and applications beyond our imagination to date. Integrating magnetic cilia with emerging technologies, such as nanomaterials, innovative 3D manufacturing, flexible electronics, soft robotics, and artificial intelligence, can lead to the creation of increasingly advanced systems boasting unprecedented functionality. With these advancements, synthetic magnetic cilia significantly contribute to shaping the future of science and engineering, leading to new discoveries and applications that can have transformative impacts.
